# Novel Designs for Application Specific MEMS Pressure Sensors

**DOI:** 10.3390/s101109541

**Published:** 2010-10-28

**Authors:** Giulio Fragiacomo, Kasper Reck, Lasse Lorenzen, Erik V. Thomsen

**Affiliations:** Department of Micro- and Nanotechnology, Technical University of Denmark, DTU Nanotech, Building 345E, DK-2800 Kgs. Lyngby, Denmark; E-Mails: lasse.lorenzen@nanotech.dtu.dk (L.L.); erik.v.thomsen@nanotech.dtu.dk (E.V.T.)

**Keywords:** micromachined, pressure, capacitive, optical, wireless, silicon, MEMS, MOEMS

## Abstract

In the framework of developing innovative microfabricated pressure sensors, we present here three designs based on different readout principles, each one tailored for a specific application. A touch mode capacitive pressure sensor with high sensitivity (14 pF/bar), low temperature dependence and high capacitive output signal (more than 100 pF) is depicted. An optical pressure sensor intrinsically immune to electromagnetic interference, with large pressure range (0–350 bar) and a sensitivity of 1 pm/bar is presented. Finally, a resonating wireless pressure sensor power source free with a sensitivity of 650 KHz/mmHg is described. These sensors will be related with their applications in harsh environment, distributed systems and medical environment, respectively. For many aspects, commercially available sensors, which in vast majority are piezoresistive, are not suited for the applications proposed.

## 1. Introduction

Commercially available micromachined pressure sensors are by a vast majority based on the piezoresistive effect, where a mechanical deformation causes a change in the electrical resistance of the sensing element which can easily be translated into a pressure signal. This principle has been tested widely in the past 40 years and is often preferred over other approaches, thanks to the fact that only standard cleanroom process such as ion implantation, oxidation and etching are involved. Compatibility to IC processes and therefore cheap process development are not the only reasons to prefer a piezoresistive sensor, also excellent linearity and simple conditioning circuits are important and quality have been the key of the success of these kind of devices. Nonetheless, strong temperature dependence of the piezoresistive coefficient π_44_, limited pressure range and scalability (due to necessity of accommodating the piezoresistors on the membrane), necessity of a DC conditioning circuit, dependence to packaging induced stress and low sensitivity have ruled out these sensors in many cases.

In this work, we will first present a touch mode capacitive pressure sensor (TMCPS) with a sensitivity of 14 pF/bar. A mathematical model that describes the behavior of TMCPS and can be used to fit experimental data will be described. Given its flat sensing surface, this sensor can easily be coated with a protective layer which makes it well suited for harsh environment applications. Furthermore, since the readout is capacitive, only an AC signal is needed and therefore the power dissipation is limited to the small amount given by the parasitic resistive contribution (*i.e.*, the connectors). We will describe a microfabrication step that can be introduced in these type of sensors in order to reduce drastically the hysteresis without compromising their sensitivity. Moreover temperature characterization of this device has been carried out showing a very low temperature dependence in both normal and touch mode.

Also the all-optical Bragg grating sensor which is presented in Section III is very well suited for harsh environments and lends itself to distributed sensing. All-optical sensors are unaffected by electromagnetic fields and noise and have low power consumption combined with good signal transmission, making them ideal for remote sensing. These properties makes them particularly well-suited for a number of demanding applications, e.g., sensing in the proximity of electrical generators, power lines and inside MRI scanners as well as sensing in fuel tanks and for oil explorations. The sensor we propose has been designed specifically for oil explorations, with focus on extremely large pressure ranges, up to 350 bar, and its frequency modulated output signal makes it the perfect candidate for distributed systems. A mathematical model which can be applied to Bragg grating optical sensors has also been developed.

Finite elements analysis has been used to characterize the last device we propose: a wireless pressure sensor for medical applications. The footprint of the sensor is small, so it can be placed inside the bladder using a catheter through the urethra. This transducer has been encapsulated in silicone in order to be implantable in the bladder tissue. A mathematical model which relates the change in pressure to a shift of the resonant frequency of the sensor has been formulated. Here, a new design based on the optimization of the fabrication parameters and the introduction of a bossed plate as the sensing element will be illustrated.

All three sensors proposed open up new possibilities for pressure measurements and have the scope to offer a solution for very sensitive pressure measurements, pressure monitoring in harsh environment or pressure monitoring inside the human body.

## 2. Capacitive Pressure Sensors in Silicon Technology

Capacitive sensing has been historically a mechanical engineering field, mainly concerning positioning, pressure and sounds measurements [1]. In the beginning of the 70’s Heerens [2] reviewed various techniques to mechanically design capacitive sensors; in the same years Jones and Richards [3] described an ultra sensitive capacitive micrometer and in 1982 Hugill [4] presented a capacitive displacement transducer. These sensors usually employed a transformer ratio bridge circuit in order to achieve the maximum possible sensitivity and a high degree of immunity to parasitic capacitance. Transformer ratio bridges, together with charge transfer circuits and more complicated methods such as switched capacitor circuits are still used to interface capacitive transducers [5–7]. While signal conditioning methods has always relayed on analog or mixed-signals electronics, the capacitive senor design, due to the needs of miniaturization, power consumption and costs issues, has shifted from being realized by bulk mechanical parts to microfabricated silicon dies. An early example of this device was fabricated for medical purposes by Frobenius *et al.* [8] in 1973. In 1986 Ko [9] compares the capacitive pressure sensor with the well established piezoresistive device. In many respects capacitive sensing results more promising than its counterpart. In 1993, Puers [10], is of the same opinion as Ko and identifies pressure as the main field of application for capacitive measurements. In 1997, Baxter [11] wrote a book which threat thoroughly the wast subject of capacitive sensing but, with respect to silicon technology, he refers to the article written by Puers four years earlier. At the same time Eaton and Smith [12] reviewed the state of the art in micromachined pressure sensor technology, in their opinion capacitive micromachined pressure sensors were still under development. Only this century saw the first commercialized devices as pointed out by Gao and Zhang [13] in 2004. Some example are the tire pressure monitoring (TPM) sensor developed by Motorola, VTI absolute pressure sensor for implantable medical devices and Vaisala barometric sensor. Finally, in 2007, Wise [14] predicts a future were MEMS of different type will be part of wireless integrated microsystems that will serve as the front-ends of information networks used in a huge variety of contexts; the capacitive pressure sensor is seen as one of the most probable candidate to be integrated in these systems.

### 2.1. Micromachined Touch Mode Capacitive Pressure Sensors: Models and Development

**Figure 1.**
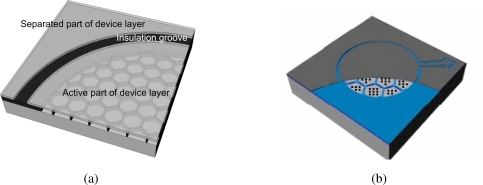
Artistic view of a touch mode capacitive pressure sensors based on an array of capacitive elements. **(a)** Artistic view of the old design, the membrane (active area) has been made transparent in order to show the support structure. **(b)** Artistic view of the new design, part of the membrane has been removed in order to show the nanopillars structure.

A micromachined capacitive pressure sensor which allows the sensing element to flatten onto an insulation layer deposited on the bottom electrode is known as touch mode capacitive pressure sensor (TMCPS). The TMCPS was firstly introduced in 1990 by Ding *et al.* [15], while an array of capacitive sensing elements in parallel was presented in the work of Dudaicevs *et al.* [16] in 1994. We have developed a TMCPS which is made of an array of elements supported by a SiO_2_ honeycomb structure. The fabrication process, where the top plate is given by the device layer of a silicon on insulator wafer, was previously described by Pedersen *et al.* [17]. In Figure 1 an artistic view of the sensor is shown; the old design, Figure 1(a), which has a flat substrate surface is compared to the new solution, Figure 1(b), where part of the membrane has been removed in order to make the newly introduced nanopillars structure visible.

This type of sensor has the advantage of eliminating interconnection between elements, thus minimizing the active area and the parasitic capacitance. Furthermore, its flat sensing surface is well suited for coating with a corrosion resistant layer, thus making this sensor a good candidate for harsh environment applications where also a low power consumption is needed.

Moreover, an analytical solution for the deflection of the membrane in all the operation regimes has been found and used to fit the experimental data [18]. In normal mode, *i.e.*, when the maximum deflection of the membrane is less than its distance from the substrate, the governing equation for the deflection, *w*, as a function of the radial distance, *r*, and the pressure, *p*, of a clamped circular isotropic plate is well known [19]

(1) $$w(r,p)=\frac{{\mathrm{pa}}_{0}^{4}}{64D}{\left(1-{\left(\frac{r}{a_{0}}\right)}^{2}\right)}^{2}$$

where *a*_0_ is the radius of the plate and *D* is the flexural rigidity of the plate given by

(2) $$D=\frac{{\mathrm{Et}}^{3}}{12\left(1-\nu^{2}\right)}$$

**Figure 2.**
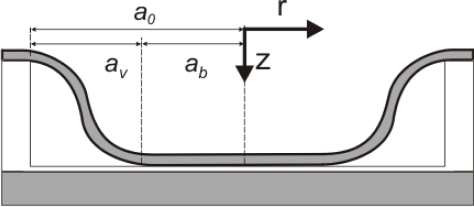
Schematic of the touch mode regime. The plate comes into contact with the insulation layer deposited on the substrate. The touching area and the suspended area of the plate are defined by the pressure dependent variables *a_b_*(*p*) and *a_v_*(*p*) which are linked by the radius of the plate *a*_0_. A polar coordinate system with the origin placed in the center of the unloaded plate has been used. The center deflection of the plate is measured along the negative z direction.

It will be shown that, in normal mode operations, the sensor proposed can be modeled under the assumption of linear elasticity and therefore described by Equation (1). A good approximation for the shape of the plate when working in touch mode (see Figure 2) is instead given by

(3) $$w\left(r,p\right)=\{\begin{array}{cc} g & 0<r<a_{b}(p)\\ g{\left(1-{\left(\frac{r-a_{b}(p)}{a_{v}(p)}\right)}^{2}\right)}^{2} & a_{b}(p)<r<a_{0} \end{array}$$

where *g* is the distance between the top electrode and the substrate at zero pressure, *a_b_* is the radius of the part of the membrane touching the substrate and *a_v_* is calculated with

(4) $$a_{v}=a_{0}-a_{b}$$

The radius of the plate touching the bottom of the cavity, *a_b_*, is a function of pressure as is also the part of the diaphragm not touching the bottom plate, *a_v_*. In the simplified model proposed, the radius *a_v_* is simply given by the center deflection being fixed at

(5) $$a_{v}(p)\equiv {\left(\frac{64\mathrm{Dg}}{p}\right)}^{1/4}$$

**Figure 3.**
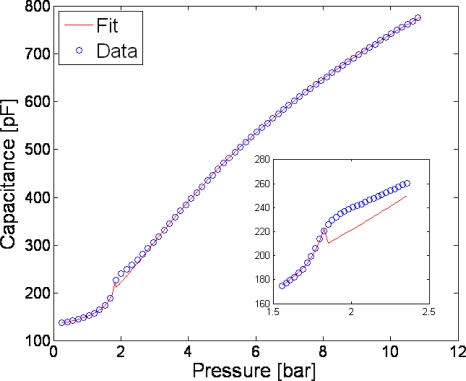
Capacitance as a function of the pressure. The analytical model (solid line) fits the experimental data (circles) and allows the extraction of important sensor parameters such as the parasitic capacitance, the insulating layer thickness and the flexural rigidity. As expected, this curve is highly nonlinear, especially in the transition region (see inlet) between normal mode and touch mode operations. The capacitance of the sensor is measured with a HP 4294A Precision Impedance Analyzer which accuracy is better than 0.1% in the range of interest. The pressure is varied with a Druck DPI 520 pressure controller which accuracy is 0.025% of the reading. Therefore the error bars cannot be seen given the high accuracy of the instrument used.

In Figure 3 it is possible to see the results given by the approximate model proposed; the blue dots represent the data points measured on the TMCPS described in [17] while the solid line is the fitting curve. From this curve parameters such as the parasitic capacitance, the flexural rigidity and the thickness of the insulation layer used to prevent short circuit when working in touch mode has been extracted. An excellent match between them and the measured ones was reported in [18] where also a solution for the integral

(6) $$C=\int_{0}^{2\pi }\int_{0}^{a_{0}}\frac{{ɛ}_{0}{ɛ}_{\mathrm{ox}}\mathrm{rdrd}\theta }{t_{\mathrm{ox}}+{ɛ}_{\mathrm{ox}}\left(g-w(r,p)\right)}$$

has been found for both operating modes.

At this point, based on the amount of research done in the field and the fact that the working principle of the TMCPS has been fully investigated and tested, we can state that this is definitely the most developed of the devices proposed. None the less, process reliability, conditioning circuit complexity and hysteresis are some of the obstacles for the commercialization of this sensor at a level comparable to its piezoresistive counterpart. We present, with the new design, a solution to the hysteresis problem of TMCPS.

**Figure 4.**
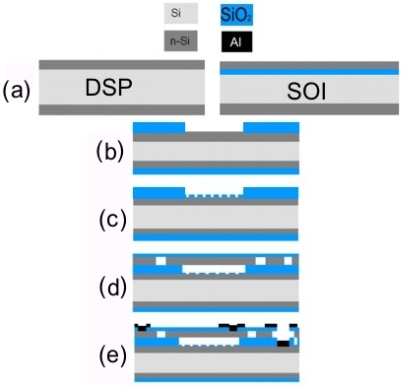
Process sequence for touch mode capacitive pressure sensors with low hysteresis. **(a)** Phosphorous doping of both the double side polished (DSP) silicon wafer and the top (SOI) wafer. **(b)** Growing of a 600 nm SiO_2_ layer and etching of the cavity. **(c)** Etching of the nanopillar structure on the bottom of the cavity and performing a second oxidation. **(d)** Etching of the insulation groove, fusion bonding and etching of the handle wafer. **(e)** Metalization of the contacts.

The process sequence for the fabrication of the new device is shown in Figure 4. Firstly a silicon and a SOI (Silicon On Insulator) wafer with 2 *μ*m are subjected to a one-hour phosphorous pre deposition in the first processing step, Figure 4 (a). A 600 nm thick oxide is grown on the silicon wafer and the cavity is etched to form the hexagonal structure mentioned earlier, Figure 4 (b). The nanopillars are then developed on the bottom of the cavities with the process described later in this section, Figure 4 (c). A second oxidation is performed to grow a 30 nm SiO_2_ layer around the pillars. This SiO_2_ layer serves as a short circuit protection when the sensor works in touch mode operation. In the next step a 10 *μ*m wide groove is etched all the way through the device layer of the SOI wafer to the buried oxide such that it will serve as an electrical insulation between the active part of the sensor, *i.e.*, the membrane area and the rest of the highly doped device layer. If these regions are not separated from each other, the area outside the membrane will add a considerable parasitic capacitance to the system. Then, the SOI wafer is turned upside down and fusion bonded with DSP wafer. Figure 4 (d) shows the result of etching the insulation groove, bonding and removing the handle wafer. Finally, the separated part of the device layer, the upper and lower electrode are contacted by means of Al evaporation, Figure 4(e).

**Figure 5.**
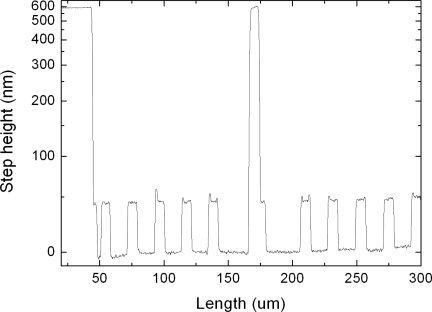
Profilometer measurement of the pillar structure etched on the bottom of the cavities. The 50 nanometer pillars are measured inside the walls of the honeycomb structure which height is roughly 600 nm.

It must be noticed that, in order to reduce the amount of hysteresis, whether due to stiction forces or to poor quality of the insulating layer, a reduction in the contact area between the top electrode and the insulation layer is needed. Two different types of corrugation can serve this purpose, a definite one obtained with a lithographic step or a random corrugated surface obtained without the use of a mask. Even though the first option requires an extra mask step (illustrated in Figure 4(c)), it must be preferred if the process involve fusion bonding of two wafers in order to keep a sufficiently smooth bonding surface. Furthermore, in the first case, the lithography limits when defining structures in a cavity (*i.e.*, with a proximity distance between the mask and the photoresist) must be considered. Experimental results has proven to be extremely difficult to approach the theoretical critical dimension, *CD*, given by [20]

(7) $$\mathrm{CD}\equiv \frac{2}{3}\sqrt{\lambda \left(d-\frac{t_{r}}{2}\right)},$$

where λ is the wavelength of the source used, *d* is the proximity distance between the mask and the photoresist and *t_r_* is the photoresist thickness. In our case, with a photoresist thickness of 1.5 *μ*m, a wavelength of 365 nm and a proximity distance equal to the gap distance between the two electrodes (600 nm) the theoretical limit was calculated to be 1.3 *μ*m. However, to achieve good results pillars of 6 *μ*m radius and around 50 nm height were fabricated with an isotropic SF6/O2 plasma etch (STS ICP Advanced Silicon Etcher). Figure 5 shows a contact profilometer (Dektak8) measurement of the nanopillars structure fabricated on the bottom of a 600 nm cavity etched in SiO_2_

**Figure 6.**
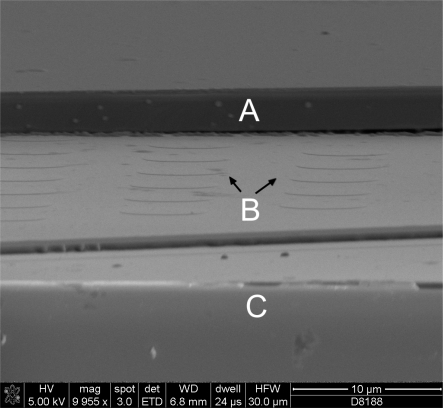
A SEM image of a fabricated chip. Part of the top plate (A) has been removed in order to see the underlying pillar structure (B) which has been fabricated on the bottom of the cavities etched in the silicon wafer (C).

A SEM image of a TMCPS fabricated with the pillar structure and diced afterwards is shown in Figure 6.

**Figure 7.**
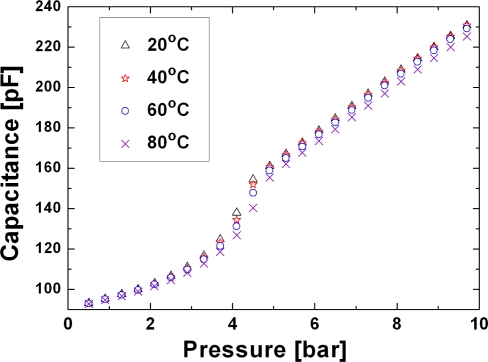
Measured capacitance as a function of the pressure at varying temperatures. From these curves the relative pressure coefficient in the different operating modes has been extracted. The capacitance of the sensor is measured with a HP 4294A Precision Impedance Analyzer which accuracy is better than 0.1% in the range of interest. The pressure is varied with a Druck DPI 520 pressure controller which accuracy is 0.025% of the reading. Therefore the error bars cannot be seen given the high accuracy of the instrument used.

**Figure 8.**
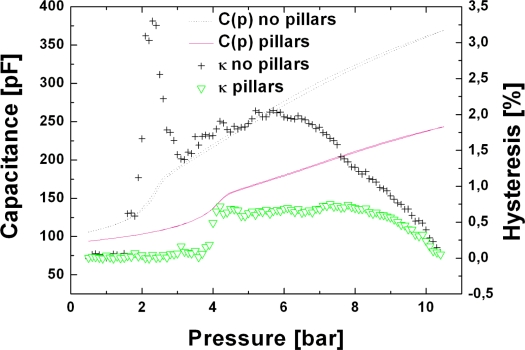
Hysteresis, *κ*, comparison between two chips of the same batch, one of them fabricated with the pillar structure (black crosses), the other without (green triangles). Capacitances curves have been added to show qualitatively the hysteresis of the sensors.

In Figure 7 capacitance as a function of the pressure at different temperature is reported, from these curves 14 pF/bar sensitivity in touch mode (where there is a nearly linear relation) was calculated. Furthermore, a relative temperature coefficient, *α*, of 0.008 %/°C, 0.023 %/°C and 0.044 %/°C have been calculated near 40 °C, 60 °C and 80 °C using

(8) $$\alpha \equiv \frac{C_{T}-C_{T_{0}}}{C_{T_{0}}(T-T_{0})}\cdot 100$$

where *C_T_* is the output signal at the temperature *T* and *C*_*T*_0__ is the output signal at room temperature, *T*_0_. Pressure cycles (where the pressure has been risen up to 10 Bar and lowered back to 500 mbar) have been carried on in order to evaluate the hysteresis, *κ*, of the sensor as

(9) $$\kappa \equiv \frac{C_{\mathrm{down}}(p)-C_{\mathrm{up}}(p)}{C_{\mathrm{up}}(p)}\cdot 100$$

where *C_down_*(*p*) is the value of the capacitance when the pressure is lowered and *C_up_*(*p*) is the value of the capacitance when the pressure is raised. In Figure 8 a hysteresis comparison between a sensor fabricated without the nanopillars structure and one were the nanopillars were added in the process flow is presented. In the letter case, the hysteresis was reduced to less than 1% in the entire pressure range. Moreover the hysteresis peak at touch point has been eliminated. These new results bring the TMCPS closer to the actual needs required for its commercialization.

## 3. Optical Pressure Sensors

Optical pressure sensors can in general be divided by detection method (amplitude or frequency modulated) and technology (electro-optical or all-optical design). Since amplitude modulation can be an on/off-signal, the sensitivity can be very high as in [21], where cantilever deflections of 10 pm is detected. However, an on/off-signal is not very well suited for multiplexing as each sensor requires its own transmission line. The sensitivity of frequency modulation is limited by the resolution of the readout system and while several frequency modulated optical sensor systems have been studied, the sensitivity is in general not as good as what can be obtained in amplitude modulated sensors. The choice of technology is a trade-off between the high flexibility and sensing capabilities of electronics and the elimination of parasitic capacitance, extremely low transmission loss and immunity to electromagnetic interference of all-optical sensors. Several designs for optical pressure sensors have been suggested in literature, though with no or only very limited commercialization. While some of these sensors are made using III–IV technology [29], the flexibility of silicon technology makes this a more obvious choice. An example of an electro-optical amplitude modulated pressure sensor has been given by Hall *et al.* [28] which combines a vertical-cavity surface-emitting laser (VCSEL), a membrane with integrated diffraction grating and photodetectors into a microphone capable of detecting down to 24 dB(A). Even though electro-optical designs show very good performance, they have none of the advantages that optical sensors have compared to electrical sensors (e.g., immunity to electromagnetic fields, low signal transmission loss and no risk of short-circuits), and are thus in direct competition with electrical sensors. In the domain of all-optical pressure sensors, the most prominent designs are the Fabry-Perot cavity [26] and the Mach-Zender interferometer [25,27] pressure sensors. In Fabry-Perot pressure sensors the deformation of a membrane causes a change in the width of a Fabry-Perot cavity which again changes the period and amplitude of the interference fringes. Depending on membrane size this can be a relatively sensitive design and using sensors with different cavity lengths, also suited for multiplexing. The Mach-Zender design utilizes the phase shift between a waveguide crossing a deflected membrane and a reference waveguide to measure pressure. The sensitivity of Mach-Zender sensors can be very high [22], but they are not easily applicable to distributed sensing systems. A common advantage to these two designs is that both are passive components *i.e.*, there are no power requirements for the sensor itself.

A closely related alternative to the Fabry-Perot sensor is the Bragg grating sensor, which creates a change in wavelength due to deformation of a waveguide with integrated Bragg grating. The Bragg grating sensor is also all-optical and frequency modulated and therefore has all the same inherent characteristics as the Fabry-Perot sensor. Contrary to the Fabry-Perot sensor, the Bragg grating sensor does not have multiple material interfaces in the light path, which reduces loss due to Fresnel reflection and it can be structured more flexible, allowing for mechanical force amplification. An example of a Bragg grating sensor is the fiber Bragg grating sensors (FBGs) [23], where fibers with UV-written gratings act as sensing elements. Fiber Bragg grating pressure sensors with detection limits of 0.36 kPa have been achieved [24], however, using MEMS technology, much better control of material and structural properties than what can be achieved in fiber technology would allow for much higher sensitivities [30].

### 3.1. All-optical MOEMS Bragg Grating Pressure Sensor

**Figure 9.**
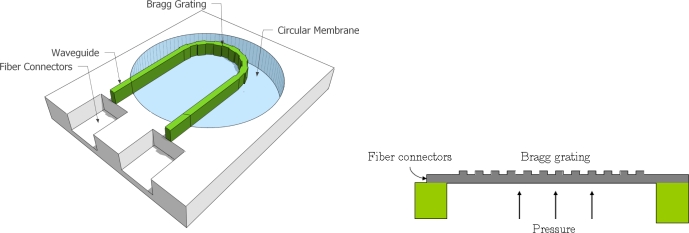
The optical pressure sensor, which 3D sketch (left figure) and cross section (right figure) are shown, consists of a circular plate with a waveguide on top. A Bragg grating is integrated into the half-circle of the waveguide. When the plate deforms the tangential strain in the grating causes a uniform change in grating period.

**Figure 10.**
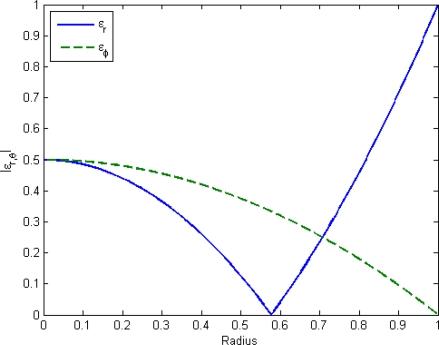
The radial and tangential strain in a circular plate in arbitrary units.

It is in many situations in oil exploration critical to obtain real-time measurements of the pressure at different locations several kilometers under ground, far from the actual readout point. Also pressure and temperature at these depths can be very high. Electrical sensors are not well suited for this scenario as as they in general do not lend themselves towards remote distributed sensing due to transmission loss and parasitic capacitances and furthermore they carry the risk of a potential short circuit causing ignition, which could be fatal in this environment. Here we present an all-optical Bragg grating sensor for high pressure and remote distributed sensing. The basic principle behind Bragg grating sensors is mechanically induced modulations of the period of a Bragg grating, causing a change in the wavelength of the reflected wave from the Bragg grating. The Bragg wavelength, λ*_B_*, is expressed through the equation

(10) $$\lambda_{B}={2n}_{\text{eff}}\Lambda$$

where *n*_eff_ is the effective refractive index and Λ is the period of the Bragg grating. The relative change in wavelength of such a sensor is given by

(11) $$\frac{{\Delta \lambda }_{B}}{\lambda_{B}}=(\alpha +\zeta )\Delta T+(1-p_{e})\varepsilon_{l}$$

where *α* is the thermal expansion coefficient of the part of the waveguide containing the grating, *ε_l_* is the longitudinal strain, *ζ* is the thermooptic coefficient, *p_e_* is the photoelastic coefficient for longitudinal strain and Δ*T* is the temperature change. Hence, the wavelength change is proportional to both temperature and the refractive index change due to strain. We will assume constant temperature, that the minimum detectable wavelength shift is 1 pm and that the Bragg wavelength is 1550 nm, hence the strain should be larger than approximately 10^−6^. Considering a circular plate with a waveguide with integrated Bragg grating on top and under uniform load, *p*, see Figure 9, the strain components in polar coordinates, *r*, θ, are [31]

(12) $$\varepsilon_{r}=-\frac{\sigma_{r}-{\nu \sigma }_{\theta }}{E}=-\frac{3}{8}\frac{(3r^{2}-a^{2}+\nu^{2}a^{2}-3r^{2}\nu^{2})p}{h^{2}E}$$

(13) $$\varepsilon_{\theta }=-\frac{\sigma_{\theta }-{\nu \sigma }_{r}}{E}=-\frac{3}{8}\frac{(r^{2}-a^{2}+\nu^{2}a^{2}-r^{2}\nu^{2})p}{h^{2}E}$$

where *ν* is the Poisson ratio, *σ* is the stress, *E* is Young’s module, *a* is the plate radius and *h* is the plate thickness. The two strains have been plotted in Figure 10.

Since the radial strain is not constant the Bragg grating would experience a non-uniform strain if it was place radially on the plate, causing lower reflection and broader reflection peak. However, while the tangentially strain is generally lower than the radial strain it would give a uniform strain in the Bragg grating was it to be placed along this direction. Since the tangential strain is monotonically decreasing with *r*, the grating should be placed as close to the center of the plate as possible, while the waveguide bending loss is kept at a minimum. The grating will still experience the radial strain, but only at a perpendicular angle. Due to the finite width of the waveguide there will be a difference in tangential strain across the waveguide. For typical waveguide widths, this will however be negligible. The minimum radius of curvature for which the waveguide can still carry a mode is [32]

(14) $$R_{\min}=\frac{\lambda }{8}\frac{1}{\sqrt{\frac{n_{\mathrm{core}}^{2}}{n_{\mathrm{cladding}}^{2}}-1}-{\text{cos}}^{-1}\left(\frac{n_{\mathrm{cladding}}}{n_{\mathrm{core}}}\right)}$$

**Figure 11.**
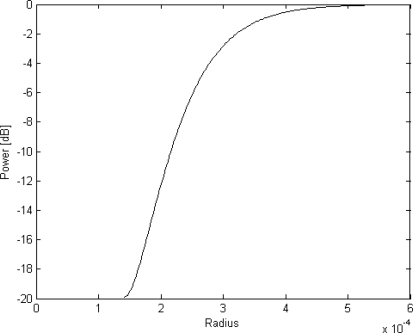
The power through an half circumference shaped 2 *μ*m wide waveguide as a function of its radius.

This puts a theoretical maximum limit on the sensitivity of this device. Assuming that the core is made of SiON (*n_core_* = 1.47) and that the cladding is made of SiO_2_ (*n_cladding_* = 1.45) the minimum radius is *R_min_* = 128 *μ*m. The power transmitted through this waveguide as function of its radius of curvature [32] is shown in Figure 11.

**Figure 12.**
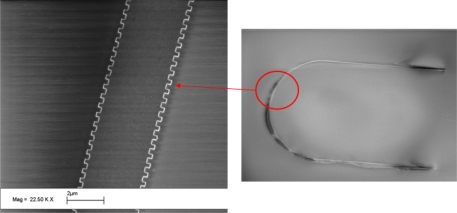
SEM image of circular plate pressure sensor and waveguide with integrated Bragg grating.

For a loss of less than 0.5 dB, the radius should be larger than 405 *μ*m. From equations 12 and 13 the two unknowns, *a* and *h*, fulfilling the requirements of pressure range, bending loss and max pressure, *i.e.*, |ε(*r* = *a, p* = *p_max_*, *r* = *R_m_in*)|≤ σ*_safe_*/*E* and ε_θ_(*r, p_min_*, *r* = *R_m_in*) ≥ ε*_min_*, can be found as

(15) $$a=\sqrt{\frac{p_{\mathrm{safe}}p_{\min}}{p_{\mathrm{safe}}p_{\min}+2\varepsilon_{\min}Ep_{\max}}}R_{\min}$$

(16) $$h=\sqrt{\frac{3p_{\min}p_{\max}\left(1-\nu^{2}\right)}{8\varepsilon_{\min}Ep_{\max}+4\sigma_{\mathrm{safe}}p_{\min}}}R_{\min}$$

where σ*_safe_* is the maximum allowable stress, *p_max_* and *p_min_* are the maximum and minimum pressure the sensor should be able to detect, respectively and *ε_min_* is the minimum detectable strain change. In oil exploration situations pressures up to 350 bar can occur. If we assume a pressure range from 0 to 350 bar (*p_max_* = 350 bar) with a resolution of *p_min_* = 1 bar, a maximum allowable stress of *σ_safe_* = 500 MPa, minimum strain of *ε_min_* = 10^−6^, a silicon membrane with *E* = 170 GPa and *ν* = 0.28, one obtains *h* = 102 *μ*m and *a* = 464 *μ*m. A SEM image of a sensor realized using the previous design rules is seen in Figure 12. The Bragg grating is located in the half circular part of the waveguide while the fiber connectors are located at the two ends of the waveguide. The waveguide and Bragg grating are made using a combination of deep reactive ion etching (DRIE) and electron beam lithography.

By using different period in each optical sensor in combination with low loss optical fibers, several sensors can be multiplexed on one single fiber transmission line. Also the small form factor (approx 2 × 2 mm^2^) and compatibility with high pressure and harsh environments make this sensor ideal for a number of applications, specifically underground oil exploration. While the temperature dependence has been neglected so far, any temperature related measurement errors can easily be compensated by integrating a pressure insensitive Bragg grating (*i.e.*, placed outside the membrane) and measuring the differential wavelength shift. For zero strain, the two reflection peaks will shift equally in wavelength when subjected to temperature changes. For non-zero strain, the two peaks will separate proportional to the strain, hence temperature and strain can be measured uncoupled.

## 4. Wireless Pressure Sensors in Silicon Technology

Wireless sensing of physical and chemical parameters is highly desirable when physical access is limited, e.g., devices for the biomedical industry and for hazardous environments [33,34]. Wireless sensing can be achieved both in an active or in a passive way. An active device has to be implemented with a microchip powered by a battery or inductively coupled power supply to make measurements [35,36], while a passive wireless sensor can be made both with Surface Acoustic Waves (SAW) or with LC resonators [33,37]. These can be measured remotely without any implemented power supply, giving passive devices the possibility of unlimited lifetime. The drawback is a limited interaction distance. The wireless development is closely linked to the medical industry since it opens new possibilities for monitoring of human parameters inside the body. In 1967 Collins *et al.* [33] made an intraocular pressure sensor based on the LC resonance method with a sensitivity of 1 MHz/mmHg. As the IC technology was developed it gave engineers a toolbox for developing more compact systems. In 1998 Park *et al.* [38] made a wafer level hermetically sealed wireless pressure sensor with an integrated LC resonator in bulk micro machining, where electroplating, anodic bonding and an implanted boron etch stop were used to achieve a footprint of 3 × 3 × 1.6 mm. The sensitivity was estimated to 2 MHz/mmHg with a resonance frequency of approximately 500 MHz. In 2001 Akar. *et al.* [39] presented a batch fabricated sealed pressure sensor with a measured sensitivity of 120 kHz/mmHg in a pressure range of 0–50 mmHg. The development has continued to produce passive wireless sensors for measuring the cerebral ventricle pressure [40] and a commercially CardioMEMS product to measure aneurysm sac pulse pressure [41].

### 4.1. Wireless Implantable Pressure Sensor

An application for passive wireless pressure sensor is to measure the pressure inside the urinary bladder, to monitor it for patients with urinary incontinence [42]. Urinary incontinence is a common problem but it is often concealed and associated with great discomfort [42]. Sensing the pressure in the urinary bladder can, via a feedback stimulation system, control the bladder, hence give control over the involuntarily excretion of urine as is characteristic for urinary incontinence [43]. Conventional sensing systems have been based on wired pressure sensors with the wires penetrating the bladder wall, causing undesirable side effects [44]. In these systems a wireless pressure sensor is designed to make reliable measurements without damaging the tissue inside the bladder.

In the medical industry new products are under heavy restrictions when it comes to lifetime and choice of material. This limits the sensor in many different ways, e.g., the device has to be biological compatible, the form factor has to be small and the design has to take diffusion and mechanical fatigue into account. From these limitations a design has been made to accommodate the different restrictions.

**Figure 13.**
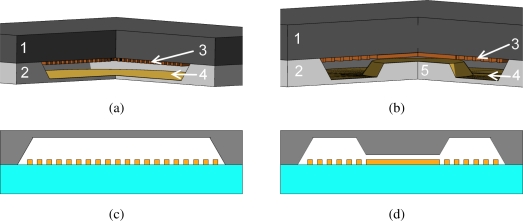
The figures shows the old and the new design of the passive bladder pressure sensor. The bottom plate and membrane are fabricated in silicon, 2, and the top plate is a glass substrate, 1. The inductor is electroplated copper, 3, and the opposite capacitor plate is made on the pressure sensitive membrane with a deposited gold film 4. **(a)** A 3D sketch of the old design. Notice that the copper coil, 3, continues to the center of the sensor. The top plate, 1, is a glass substrate. The bottom plate, 2, is a silicon substrate with a etched membrane covered with a gold layer, 4. **(b)** A 3D sketch of the new design. Notice the bossed membrane in the the center of the sensor, 5, and the electroplated copper coil, 3, that is solid in the center. The membrane deflects in the region, 4, that is situated between the bossed structure, 5, and the silicon substrate, 2. **(c)** A 2D sketch of the old design. **(d)** A 2D sketch of the new design.

The sensing principle is capacitive, where the varying capacitance is included in a LC circuit. The capacitor plates are a circular membrane coated with metal on one side, and an electroplated copper spiral on a glass substrate on the other side. Furthermore, the spiral also functions as the inductor for the LC circuit. Combining the coil as one of the capacitor plates enables the device to become smaller and more compact compared to similar designs. The first conceptual design was a simple structure, made by a KOH etched membrane, copper electroplated coil, e-beam evaporated capacitor plate and anodic bonding, see Figure 13(a). To improve the design, simulation of a bossed membrane is used to optimize sensitivity, Q-factor and resonance frequency. The new design can be seen in Figure 13(b). According to classical electrodynamics this circuit has angular resonance frequency, *ω_res_*, and a Q-factor, *Q*, that can be calculated knowing the inductance, *L*, the capacitance, *C*, and the resistance, *R*, of the circuit

(17) $$\omega_{\mathrm{res}}=\frac{1}{\sqrt{\mathrm{LC}}}$$

(18) $$Q=\frac{1}{R}\sqrt{\frac{L}{C}}$$

**Figure 14.**
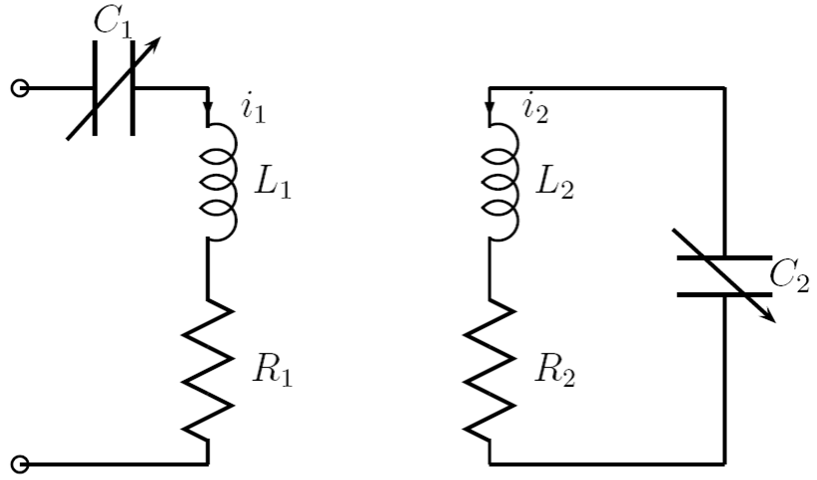
Equivalent circuit diagram of a transformer circuit where the oscillation circuit on the primary side, made up by *C*_1_ and *L*_1_, can be tuned to that of the secondary side *C*_2_ and *L*_2_.

To measure this angular resonance frequency through an external measuring circuit, the circuit is coupled to the sensor through a mutual inductance using a tuned transformer coupling, see Figure 14. Here the resonance of the sensor is reflected in the external measuring circuit through the coupling constant, *k*, and the external coil, *L*_1_. The impedance of the system seen from the external terminals is given by

(19) $$Z(j\omega )=R_{1}+j\frac{{\left(\frac{\omega }{\omega_{01}}\right)}^{2}+1}{\omega C_{1}}+j\omega L_{1}k^{2}\frac{{\left(\frac{\omega }{\omega_{02}}\right)}^{2}}{j\frac{\omega }{Q_{2}\omega_{02}}-{\left(\frac{\omega }{\omega_{02}}\right)}^{2}+1},$$

where *Q*_2_ is the Q-factor of the sensor, *j* is the imaginary constant, *ω* is the angular frequency, *ω*_01_ and *ω*_02_ are the resonance angular frequencies of the external system and the sensor, respectively.

**Figure 15.**
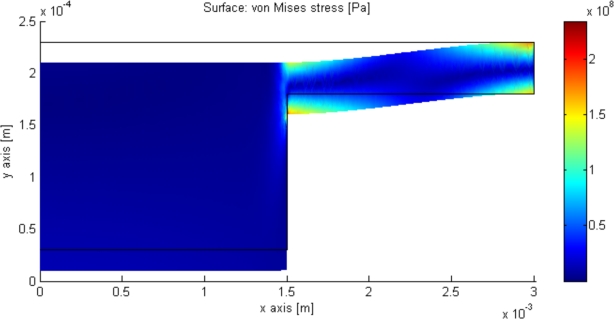
A structural mechanical simulation made in COMSOL for 1 bar pressure is shown. The black outline is the unstrained structure. Notice that the largest stress is located in the thin membrane, and the bossed structure is only moving vertically movement according to the center defection. Axial symmetry is employed in this simulation, the y-axis is considered the axis of rotation.

**Figure 16.**
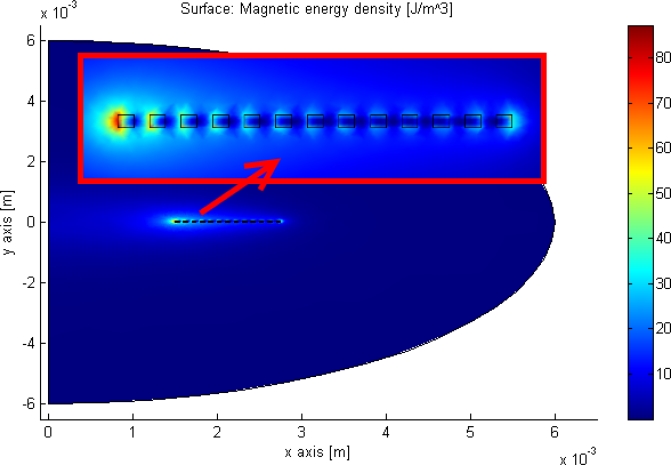
A static electrical simulation made in COMSOL is shown. The inductor is enclosed in an air domain to encapsulate all of the electromagnetic field. The cutout section in the red box shows a zoom of the inductor, that is approximated with concentric rings. Axial symmetry is employed in this simulation, the y-axis is considered the axis of rotation.

Scaling the sensor implies scaling the radius of the sensor, as the inductance of a spiral is proportional with the radius cubed, whereas the capacitor is proportional to the radius squared. This implies that the Q-factor of the sensor increases with its dimensions. This also gives rise to an increase in the impedance of the system, see Equation 19. Unfortunately, due to the biological application of the device the size is restricted to avoid excessive discomfort and side effects. A trade off between these has to be made. The old design is 6 × 6 mm wide and has a thickness below 1 mm, the same outer dimensions as the new design. This is small enough to avoid disturbing the bladder, and large enough to produce a measurable signal. The inductor and capacitor are also important; since the capacitance change governs the sensitivity and the inductor governs the signal strength there is a trade-off between these as well. Finite element method (FEM) simulation is made using the commercial software COMSOL in order to optimize the sensor. Figure 15 shows a structural mechanical simulation made with the parameters for the new design shown in Table 1 at 1 bar and used to extract the deflection of the sensor. Then the parallel plate capacitor approximation is used to calculate the capacitance. The quasi-static magnetic module in COMSOL, see Figure 16, is then used to simulate the inductance by solving the magnetic vector potential using Maxwell equations. The inductor is enclosed in a sphere with radius twice as large as the inductor radius, in order to encapsulate all of the electromagnetic fields. These two FEM simulations are programmed to investigate the resonance frequency, sensitivity and Q-factor as a function of the design parameters shown in Table 1. The simulation are made for an absolute pressure range of 0 to 330 mmHg, which for safety reasons is 10 times higher than the pressure range of interest.

**Figure 17.**
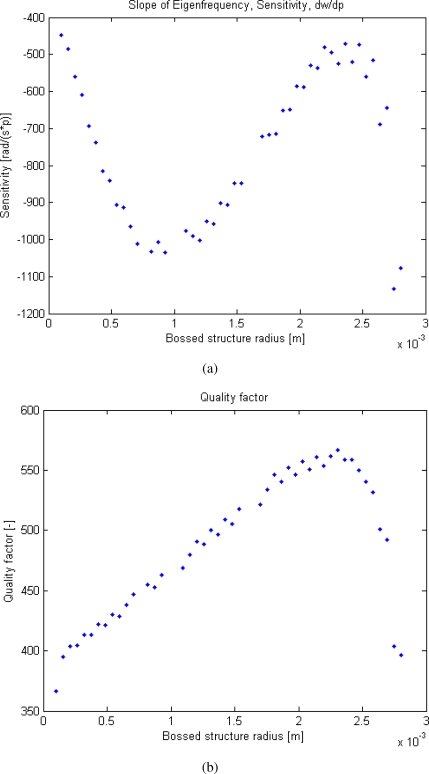
The figures shows simulations of a bossed membrane structure with parameters: gap distance at vacuum = 80 *μ*m, radius of membrane = 3 mm, spiral width = spiral spacing = spiral thickness = 50 *μ*m and thickness of bossed structure = 100 *μ*m. The design parameters are not used because it would limit the range, and in these simulations the general behavior is investigated. The internal radius of the bossed structure is varied from 0 to 3 mm. **(a)** Sensitivity *vs.* boss radius. An optimal internal radius of the bossed membrane is around 1.2 mm. **(b)** Q-factor *vs.* boss radius. A clear peak is seen at approximately 2.4 mm.

From the simulations it can be seen that the sensitivity is monotonically increasing as the bossed structure gets thicker and membrane gets thinner and plate distance decreases, whereas the Q-factor is monotonically decreasing. More interesting are the variations of the radius of the bossed structure on the new design, see Figure 17(a). Here the optimal radius, in terms of sensitivity, can be read from the numerical simulations shown on the graph and is approximately 1.2 mm, whereas the Q-factor, see Figure 17(b), is increasing up to a radius of approximately 2.4 mm. It can be seen that a higher sensitivity, if compared to the old design, can be reached implementing a radius of approximately 1 to 1.5 mm, still keeping a satisfactory Q-factor. According to the simulation made with the new design parameters shown in Table 1 a sensitivity of 650 kHz/mmHg, Q-factor of 240 and a resonance frequency of 86 MHz can be reached compared to the old design which had a sensitivity of 30 kHz/mmHg, Q-factor of 150 and a resonance frequency of 63 MHz.

From the work done it has been shown that a more sensitive sensor can be produced by adding a bossed structure and still keeping the original features of an hermetically sealed small sensor. The design modifications introduced lead to a more complicated fabrication process but, with the improvements on the performances of the sensor, this is of secondary importance. Finally, the simulation tool presented allows a fast evaluation of the impact on the performances of these sensors given by a modification of their fabrication parameters.

## 5. Discussion and Conclusions

A TMCPS, an all-optical pressure sensor and a wireless pressure sensor have been presented. Their design has been validated either with an analytical or with a finite element model. Furthermore, one or more fields of application for each sensor have been identified. The possibility of using them in harsh environments, as well as low power consumption (especially compared to the piezoresistive pressure sensor), are the intrinsic common features of the devices proposed. None the less, while the TMCPS is in a more advanced stage of development and has been characterized with respect to temperature, the MOEMS pressure sensor has the unique feature of being immune to electromagnetic interference and is also expected to have a good temperature insensitivity. The wireless device, being a passive transducer, can be used for applications where, obviously, the other two solutions cannot be used.

Table 2 summarizes all these aspects and specifies the pressure ranges and sensitivities of the three designs. An experimental validation of the designs proposed was possible only for the TMCPS. However the guidelines given for the fabrication of all three type of sensors lead to a step forward in the development of pressure sensors for application specific purposes.

## Figures and Tables

**Table 1.** In this table the parameters used for the simulations are shown, both for the old and the new design.

**Parameter**	**Old Design**	**New Design**
Membrane thickness	70 *μm*	50 *μm*
Gap distance at vacuum	40 *μm*	30 *μm*
Radius of the membrane	3 *mm*	3 *mm*
Spiral width	50 *μm*	50 *μm*
Spiral spacing	50 *μm*	50 *μm*
Spiral thickness	50 *μm*	50 *μm*
Radius of bossed structure	-	1.5 *mm*
Thickness of bossed structure	-	150 *μm*

**Table 2.** Mathematical model, characteristics and applications for the three designs proposed.

**Sensor type**	**Model**	**Pressure range**	**Sensitivity**	**Technology status**	**Applications**
TMCPS	analytical	0–10 bar	14 pF/bar	prototyping	harsh environment, low power consumption
All-optical pressure sensor	analytical	0–350 bar	1 pm/bar	proof of concept	harsh environment, low power consumption, distributed systems
Wireless passive pressure sensor	FEM	0–330 mmHg	650 KHz/mmHg	proof of concept	harsh environment, low power consumption, medical
